# Health-focused conversational agents in person-centered care: a review of apps

**DOI:** 10.1038/s41746-022-00560-6

**Published:** 2022-02-17

**Authors:** Pritika Parmar, Jina Ryu, Shivani Pandya, João Sedoc, Smisha Agarwal

**Affiliations:** 1 The Johns Hopkins University Krieger School of Arts and Sciences, Baltimore, MD USA; 2 Department of International Health, The Johns Hopkins University Bloomberg School of Public Health, Baltimore, MD USA; 3 Department of Technology, Operations and Statistics, New York University Stern School of Business, New York City, NY USA

**Keywords:** Quality of life, Patient education, Lifestyle modification, Public health

## Abstract

Health-focused apps with chatbots (“healthbots”) have a critical role in addressing gaps in quality healthcare. There is limited evidence on how such healthbots are developed and applied in practice. Our review of healthbots aims to classify types of healthbots, contexts of use, and their natural language processing capabilities. Eligible apps were those that were health-related, had an embedded text-based conversational agent, available in English, and were available for free download through the Google Play or Apple iOS store. Apps were identified using 42Matters software, a mobile app search engine. Apps were assessed using an evaluation framework addressing chatbot characteristics and natural language processing features. The review suggests uptake across 33 low- and high-income countries. Most healthbots are patient-facing, available on a mobile interface and provide a range of functions including health education and counselling support, assessment of symptoms, and assistance with tasks such as scheduling. Most of the 78 apps reviewed focus on primary care and mental health, only 6 (7.59%) had a theoretical underpinning, and 10 (12.35%) complied with health information privacy regulations. Our assessment indicated that only a few apps use machine learning and natural language processing approaches, despite such marketing claims. Most apps allowed for a finite-state input, where the dialogue is led by the system and follows a predetermined algorithm. Healthbots are potentially transformative in centering care around the user; however, they are in a nascent state of development and require further research on development, automation and adoption for a population-level health impact.

## Introduction

In recent years, there has been a paradigm shift in recognizing the need to structure health services, so they are organized around the individuals seeking care, rather than on the disease^1,2^. Integrated person-centered health services require that individuals be empowered to take charge of their own health, and have the education and support they need to make informed health decisions^3^. Over the last decade, the internet and the use of health apps have emerged as critical spaces where individuals are accessing health information. In the U.S., over 70% of the population uses the Internet as a source of health information^4^. A 2017 study in Sub-Saharan Africa, reported that 41% of Internet users used the Internet for health information and medicine^5^, highlighting the value of the internet as a global health information source. Concurrently, there has also been a proliferation of health-related apps, with an estimated 318,000 health apps available globally in 2017^6^. To facilitate two-way health communication and center care around the individual user, apps are integrating conversational agents or “healthbots” within the app^7,8^.

Healthbots are computer programs that mimic conversation with users using text or spoken language^9^. The advent of such technology has created a novel way to improve person-centered healthcare. The underlying technology that supports such healthbots may include a set of rule-based algorithms, or employ machine learning techniques such as natural language processing (NLP) to automate some portions of the conversation. Healthbots are being used with varying functions across a range of healthcare domains; patient-facing healthbots^9^ are largely focused on increasing health literacy^10^, mental health (i.e., depression, anxiety)^11–16^, maternal health^17–19^, sexual health and substance use^20^, nutrition and physical activity^21–23^, among others.

The use of healthbots in healthcare can potentially fill a gap in both the access to and quality of services and health information. First, healthbots are one way in which misinformation could be managed within the online space by integrating evidence-based informational bots within existing social media platforms and user groups. In a 2018 American Association of Family Physicians survey of family practitioners, over 97% of providers noted discussions with patients regarding inaccurate or incorrect health information from the Internet^24^. Second, healthbots can also be used to triage patients presenting with certain symptoms, as well as to provide additional counseling support after a clinical encounter, thereby reducing the burden on the healthcare system, while also prioritizing patient experience. This is increasingly important given the global shortage of healthcare workers, estimated at a deficit of around 18 million by 2030^25^. Healthbots can be an adjuvant to clinical consultations, serving as an informational resource beyond the limited amount of time for doctors-patient interactions^26^. A few recent studies suggest engagement with healthbots results in improvements in symptoms of depression and anxiety^11,16,27^, preconception risk among African American women^17^, and literacy in sexual health and substance abuse prevention among adolescents^20^. In addition to providing evidence-based health information and counseling, healthbots may aid in supporting patients and automating organizational tasks, such as scheduling appointments, locating health clinics, and providing medication information^28^. While more robust evaluations of the impact of healthbots on healthcare outcomes are needed, preliminary results suggest this is a feasible approach to engage individuals in their healthcare. Often, interventions that use healthbots are targeted at patients/clients, without the active engagement of a healthcare provider. As such, healthbots may also pose certain risks. Especially in cases where such interventions employ machine learning approaches, it is important to understand and monitor the measures that are taken by developers to ensure patient/client safety. Currently, there is a lack of a clear regulatory framework for such health interventions, which may pose a range of risks to users including threats to their privacy and security of healthcare information^27,29^.

While healthbots have a potential role in the future of healthcare, our understanding of how they should be developed for different settings and applied in practice is limited. A few systematic and scoping reviews on health-related chatbots exist. These have primarily focused on chatbots evaluated in peer-reviewed literature^9,27,30–33^, provided frameworks to characterize healthbots and their use of ML and NLP techniques^9,32,33^, are specific to health domains (e.g., mental health;^27,30,31^ dementia^34^), for behavior change^35^, or around the design and architecture of healthbots^9,32,36^. There has been one systematic review of commercially available apps; this review focused on features and content of healthbots that supported dementia patients and their caregivers^34^. To our knowledge, no review has been published examining the landscape of commercially available and consumer-facing healthbots across all health domains and characterized the NLP system design of such apps. This review aims to classify the types of healthbots available on the app store (Apple iOS and Google Play app stores), their contexts of use, as well as their NLP capabilities.

To facilitate this assessment, we develop and present an evaluative framework that classifies the key characteristics of healthbots. Concerns over the unknown and unintelligible “black boxes” of ML have limited the adoption of NLP-driven chatbot interventions by the medical community, despite the potential they have in increasing and improving access to healthcare. Further, it is unclear how the performance of NLP-driven chatbots should be assessed. The framework proposed as well as the insights gleaned from the review of commercially available healthbot apps will facilitate a greater understanding of how such apps should be evaluated.

## Methods

### Search strategy

We conducted iOS and Google Play application store searches in June and July 2020 using the 42Matters software. 42Matters is a proprietary software database that collects app intelligence and mobile audience data, tracking several thousand metrics for over 10 million apps^37^; it has been used previously to support the identification of apps for app reviews and assessments^38,39^. A team of two researchers (PP, JR) used the relevant search terms in the “Title” and “Description” categories of the apps. The language was restricted to “English” for the iOS store and “English” and “English (UK)” for the Google Play store. The search was further limited using the Interactive Advertising Bureau (IAB) categories “Medical Health” and “Healthy Living”. The IAB develops industry standards to support categorization in the digital advertising industry; 42Matters labeled apps using these standards^40^. Relevant apps on the iOS Apple store were identified; then, the Google Play store was searched with the exclusion of any apps that were also available on iOS, to eliminate duplicates.

Search terms were identified leveraging Laranjo et al. and Montenegro et al., following a review of relevant literature^9,32^. The search terms initially tested in 42Matters for both app stores were: “AI”, “assistance technology”, “bot”, “CBT”, “chat”, “chatbot”, “chats”, “companion”, “conversational system”, “dialog”, “dialog system”, “dialogue”, “dialogue system”, “friend”, “helper”, “quick chat”, “therapist”, “therapy”, “agent”, and “virtual assistant.” 42Matters searches were initially run with these terms, and the number of hits was recorded. The first 20 apps for each term were assessed to see if they are likely to be eligible; if not, the search term was dropped. For instance, searching “helper” produced results listing applications that provided assistance for non-healthcare related tasks, including online banking, studying, and moving residence; therefore “helper” was excluded from the search. This process resulted in the selection of nine final search terms to be used in both the iOS Apple and Google Play store: “agent”, “AI”, “bot”, “CBT”, “chatbot”, “conversational system”, “dialog system”, “dialogue system”, and “virtual assistant”. Data for the apps that were produced using these search terms were downloaded from 42Matters on July 16th, 2020.

### Eligibility criteria and screening

The study focused on health-related apps that had an embedded text-based conversational agent and were available for free public download through the Google Play or Apple iOS store, and available in English. A healthbot was defined as a health-related conversational agent that facilitated a bidirectional (two-way) conversation. Applications that only sent in-app text reminders and did not receive any text input from the user were excluded. Apps were also excluded if they were specific to an event (i.e., apps for conferences or marches).

Screening of the apps produced by the above search terms was done by two independent researchers (PP, JR) on the 42Matters interface. To achieve consensus on the inclusion, 10% of the apps (*n* = 229) were initially screened by two reviewers. The initial screening included a review title and descriptions of all apps returned by the search. This process yielded a 91% agreement between the two reviewers. Disagreements were discussed with the full research team, which further refined the inclusion criteria. Based on this understanding, the remaining apps were screened for inclusion.

All apps that cleared initial screening, were then downloaded on Android or iOS devices for secondary screening. This included as assessment of whether the app was accessible and had a chatbot function. For apps that cleared secondary screening, the following information was downloaded from 42Matters: app title, package name, language, number of downloads (Google Play only), average rating, number of ratings, availability of in-app purchases, date of last update, release date (Apple iOS only), use of IBM Watson software, Google sign-in (Apple iOS only), and Facebook sign-in (Apple iOS only). Utilization of Android permissions for Bluetooth, body sensors, phone calls, camera, accounts access, and internet as well as country-level downloads was also extracted from Google Play store only.

### Data synthesis

For data synthesis, an evaluation framework was developed, leveraging Laranjo et al., Montenegro et al., Chen et al., and Kocaballi et al.^9,32,41,42^. Two sets of criteria were defined: one aimed to characterize the chatbot, and the second addressed relevant NLP features. Classification of these characteristics is presented in Boxes 1 and 2. We calculated the percentage of healthbots that had each element of the framework to describe the prevalence of various features in different contexts and healthcare uses. Determination of the NLP components of the app was made based on the research team using the app and communicating with the chatbot. If certain features were unclear, they were discussed with the research team, which includes a conversational agent and natural language processing expert. To understand the geographic distribution of the apps, data were abstracted regarding the highest percentage of downloads for each app per country, for the top five ranking countries. Percentages were taken of the total number of downloads per app per country, which was only publicly available for the Google Play store (*n* = 22), to depict the geographic distribution of chatbot prevalence and downloads globally.

This study protocol was not registered.

Box 1 Characterization of the Healthbot App—Health Contexts and Core Features (Short Title: *Health Context and Features of the Apps*)

**Table:** 

**Context of īnteraction**	
*User* ^*a*^	Patients: The target population was patients with specific illnesses or diseases.
	Healthcare providers: The target population was healthcare providers.
	Any user: The target population was the general public including caregivers or healthy individuals.
	Undetermined: The target population of the app was not able to be determined.
*Health domain areas* ^a^	Mental health: The app was developed for a mental health domain area.
	Primary care: The app was developed for a primary care domain area, which included healthbots containing symptom assessment, primary prevention, and other health-promoting measures.
	Other: The app was developed for one of the following focus areas: Anesthesiology, Cancer, Cardiology, Dermatology, Endocrinology, Genetics, Medical Claims, Neurology, Nutrition, Pathology, Sexual Health
	Undetermined: The domain area of the app was not able to be determined.
**Theoretical or therapeutic underpinning**	Yes: The app included a theoretical or therapeutic underpinning (ex. Cognitive Behavioral Therapy (CBT), Dialectic Behavioral Therapy (DBT), Stages of Change/Transtheoretical Model).
	No: The app did not include a theoretical or therapeutic underpinning.
	Undetermined: The theoretical or therapeutic underpinning of the app was not able to be determined.
**Level of personalization** ^b^	
*Automation*	Yes: The app used personalization.
	Implicit: Information needed for personalization was obtained automatically through analysis of user interactions with the system.
	Explicit: Information needed for user models required users’ active participation.
	No: The app did not use personalization.
	Undetermined: The personalization of the app was not able to be determined.
*Target*	Individuated: The personalization was targeted at a specific individual.
	Categorical: The personalization was targeted at a group of people.
*Aspects of system*	Content: The information itself could be personalized.
	User interface: The way in which the information is presented could be personalized.
	Delivery channel: The media through which information is delivered could be personalized.
	Functionality: The functions of the app could be personalized.
**Additional engagement features**	Appointment scheduling^c^: The app included an appointment scheduling feature.
	Clinic/Services locator^c^: The app included a clinic/service locating feature.
	Create account: The app included an option to create an account for the app in order to store information for later use.
	Decision aid support^c^: The app included a decision aid support feature that advised users to seek additional medical care if appropriate.
	Embodied conversational agent/chatbot^d^: The app included an embodied conversational agent/chatbot.
	Integration of videos^c^: The healthbot integrated videos into its conversations.
	Main menu/navigation bar^c^: The app included a main menu/navigation bar.
	Push notifications/reminders to use^c^: The app included push notifications/reminders to use it.
	Required purchase to use: The app required purchase to use or offered in-app purchases.
	Redirect to doctor/therapist: The app redirected users to a doctor or a therapist.
	No additional features: The app did not include additional features.
	Undetermined: The app’s additional features were not able to be determined
	Other^e^: The app included one or more of the following features (see footnote)
**Mobile/web access** ^f^	Mobile: The app was available as an installable software via mobile devices and tablets.
	Web: The app was accessible via a web browser on laptops, desk computers, phones, tablets, etc.
	Undetermined: The app was not accessible.
**Security**	E-mail verification: The app sent a one-time password to the user’s e-mail for identity verification.
	Text verification: The app sent a one-time password to the user’s phone number for identity verification.
	Social media verification: The app provided the option to sign into a social media account for identity verification.
	Passcode to access app: A password was required to access the app.
	No security elements: The app did not contain any security elements.
	Undetermined: The security of the app was not able to be assessed.
**Privacy**	HIPAA: The app stated that it was HIPAA compliant.
	Child Online Privacy and Protection Act (COPPA): The app stated that it was COPPA compliant.
	Medical disclaimer: The app provided a medical disclaimer that it is not a substitute for care from a healthcare professional.
	Other privacy elements: The app contained one of the following privacy elements: encrypted data disclaimer, app-specific privacy policy
	No privacy elements: The app did not contain any privacy elements.
	Undetermined: The privacy of the app was not able to be assessed.

^a^(adapted from Montenegro et al.).

^b^(adapted from Kocaballi et al.).

^c^(adapted from Chen et al.).

^d^(adapted from ter Stal et al.).

^e^Other: Billing; Bluetooth Connection to Health Device; Clarity of text/images (Chen); Connect to store; Emergency Mode for Urgent Matters; Forum or Social Network (Chen); Gamification (Chen); GPS (Chen); Internal search function (Chen); Lab Results/Health History; Link to additional information; Order Tests/Medication; Tracker for food or symptoms.

^f^ (adapted from Laranjo et al.).

Box 2 Characterization of Natural Language Processing (NLP) System Design (Short Title: *NLP System Design of the Apps*)

**Table:** 

**Dialogue management** ^a^ *How the system manages the conversation between the healthbot and the user*.	Finite-state: The user is taken through a predetermined flowchart of steps and states. If the user veers from the path, the system is unable to respond.
	Frame-based: The user is asked questions and the system fills slots in a template to perform a task. If information regarding multiple slots on the template is given, the system interprets both. Thus, the dialog flow is not predetermined but rather depends on the user’s input and the additional information the system needs.
	Agent-based: This enables complex communication between the system, the user, and the application. This uses advanced statistical and AI methods to manage the conversation.
	Undetermined: The app’s dialogue management was not able to be determined.
**Dialogue interaction method** ^b^ *Which method the healthbot employs to interact with the user in the conversation*.	Fixed input: The system is responsible for managing the state of dialogue during interactions between the agent and the user. It characteristically uses "button-push" interfaces with finite responses. It typically does not involve NLP techniques.
	Basic parser: The system parses and computes the input to decide on a final reply to the user. It can only respond to basic questions.
	Semantic parser: The system uses a flexible plan so dialogue-based interaction can be dynamically calculated based on information the system gathers about the user. It can answer a wider range of questions as it is not restricted to keywords. It is also able to glean themes from past user questions and dynamically respond accordingly.
	AI generation: The system generates replies to users through machine learning algorithms or statistical approaches. It can respond to complex questions of 2–3 sentences.
	Undetermined: The app’s dialogue interaction method was not able to be determined.
**Dialogue initiative** ^a^ *Who initiates the conversation between the healthbot and the user*.	User: The user leads the conversation.
	System: The system leads the conversation.
	Mixed: Both the user and the system can lead the conversation.
	Undetermined: The app’s dialogue initiative was not able to be determined.
**Input modality** ^a^ *How the user can communicate with the healthbot*.	Spoken: The user must use spoken language to interact with the system.
	Written: The user uses written language to interact with the system.
	Visual: The user uses visual cues (e.g., graphics) to interact with the system.
	Undetermined: The app’s input modality was not able to be determined.
**Output modality** ^a^ *How the healthbot system can communicate with the user*.	Spoken: The system uses spoken language to interact with the user.
	Written: The system uses written language to interact with the user.
	Visual: The system uses visual cues (e.g., graphics) to interact with the user.
	Undetermined: The app’s output modality was not able to be determined.
**Task-oriented** ^a^ *If the healthbot system is intended for a specific task, or is intended purely for conversation*.	Yes: The system is designed for a particular task and thus engages in short conversations to determine the necessary information to accomplish this set goal.
	No: The system is not set up to fulfill a short-term goal or task.
	Undetermined: The app’s task orientation was not able to be determined.

^a^(adapted from Laranjo et al.).

^b^(adapted from Montenegro et al.).

## Results

**Fig. 1:**
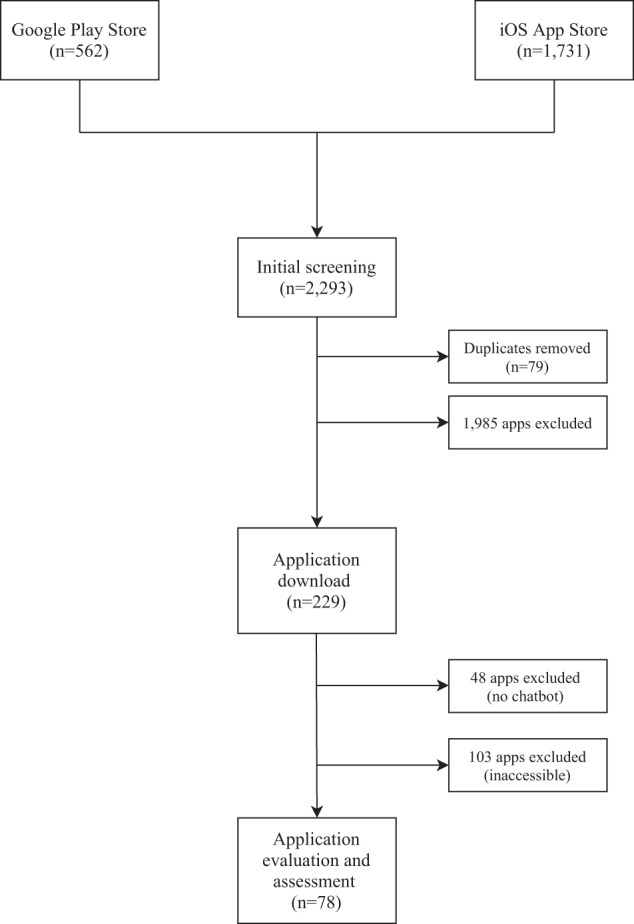
Consort Diagram. This consort diagram shows the app selection process.

The search initially yielded 2293 apps from both the Apple iOS and Google Play stores (see Fig. 1). After the initial screening, 2064 apps were excluded, including duplicates. The remaining 229 apps were downloaded and evaluated. In the second round of screening, 48 apps were removed as they lacked a chatbot feature and 103 apps were also excluded, as they were not available for full download, required a medical records number or institutional login, or required payment to use. This resulted in 78 apps that were included for review (See Appendix 1).

Twenty of these apps (25.6%) had faulty elements such as providing irrelevant responses, frozen chats, and messages, or broken/unintelligible English. Three of the apps were not fully assessed because their healthbots were non-functional.

### App characteristics and core features

The apps targeted a range of health-related goals. Forty-seven (42%) of the apps supported users in performing health-related daily tasks including booking an appointment with a healthcare provider and guided meditation, twenty-six (23%) provided information related to a specific health area including some which provided counselling support for mental health, twenty-two (19%) assessed symptoms and their severity, sixteen (14%) provided a list of possible diagnoses based on user responses to symptom assessment questions, and two (2%) tracked health parameters over a period of time. Table 1 presents an overview of other characteristics and features of included apps.

**Table 1:** Characterization of the Chatbot—Health Contexts and Core Features.

Evaluation criteria	*n* (%)
*Context of interaction*	
User (*n* = 140^a^)	
Patients	74 (52.86)
Healthcare providers	6 (4.29)
Any user	60 (42.86)
Undetermined	0 (0)
Health domain areas (*n* = 152^a^)	
Mental health	22 (14.47)
Primary care	47 (30.92)
Other^b^	83 (54.61)
Undetermined	0 (0)
*Theoretical or therapeutic framework*^c^(*n* = 79^a^)	
Yes	6 (7.59)
No	73 (92.41)
Undetermined	0 (0)
*Level of personalization*	
Automation (*n* = 78)	
Yes	47 (60.26)
Implicit	0 (0)
Explicit	47 (100.00)
No	29 (37.18)
Undetermined	2 (2.56)
Target (*n* = 47)	
Individuated	47 (100.00)
Categorical	0 (0)
Aspects of system (*n* = 48^d^)	
Content	43 (89.58)
User interface	5 (10.42)
Delivery channel	0 (0)
Functionality	0 (0)
*Additional features* (*n* = 128^a^)	
Appointment scheduling	7 (5.47)
Clinic/services locator	5 (3.91)
Create account	8 (6.25)
Decision aid support	8 (6.25)
Embodied conversational agent/chatbot	5 (3.91)
Integration of videos	6 (4.69)
Main menu/navigation bar	5 (3.91)
Push notifications/reminders to use	26 (20.31)
Required purchase to use	5 (3.91)
Redirect to doctor/therapist	6 (4.69)
No additional features	19 (14.84)
Undetermined	3 (2.34)
Other^e^	25 (19.53)
*Mobile/Web Access* (*n* = 88^a^)	
Mobile	78 (88.64)
Web	10 (11.36)
Undetermined	0 (0)
*Security* (*n* = 78)	
E-mail verification	9 (11.54)
Text verification	3 (3.85)
Social media verification	1 (1.28)
Passcode to access app	1 (1.28)
No security elements	62 (79.49)
Undetermined	2 (2.56)
*Privacy* (*n* = 81^a^)	
HIPAA	10 (12.35)
Child Online Privacy and Protection Act (COPPA)	3 (3.70)
Medical disclaimer	13 (16.05)
Other privacy elements^f^	2 (2.47)
No privacy elements	51 (62.96)
Undetermined	2 (2.47)

^a^Total sample size exceeds 78 because the healthbot can fulfill multiple categories.

^b^Other: anethesiology, cancer, cardiology, dermatology, endocrinology, genetics, medical claims, neurology, nutrition, pathology, and sexual health.

^c^Models: Cognitive Behavioral Therapy (CBT), Dialectic Behavioral Therapy (DBT), Stages of Change/Transtheoretical Model.

^d^Total sample size exceeds 47 because the healthbot can fulfill multiple categories.

^e^Other: Billing; Bluetooth Connection to Health Device; Clarity of text/images (Chen); Connect to store; Emergency Mode for Urgent Matters; Forum or Social Network (Chen); Gamification (Chen); GPS (Chen); Internal search function (Chen); Lab Results/Health History; Link to additional information; Order Tests / Medication; Tracker for food or symptoms.

^f^Other privacy elements include: encrypted data disclaimer, app-specific privacy policy.

Most apps were targeted at patients. Seventy-four (53%) apps targeted patients with specific illnesses or diseases, sixty (43%) targeted patients’ caregivers or healthy individuals, and six (4%) targeted healthcare providers. The total sample size exceeded seventy-eight as some apps had multiple target populations.

The apps targeted one or more health domain areas. There were 47 (31%) apps that were developed for a primary care domain area and 22 (14%) for a mental health domain. Involvement in the primary care domain was defined as healthbots containing symptom assessment, primary prevention, and other health-promoting measures. Additionally, focus areas including anesthesiology, cancer, cardiology, dermatology, endocrinology, genetics, medical claims, neurology, nutrition, pathology, and sexual health were assessed. As apps could fall within one or both of the major domains and/or be included in multiple focus areas, each individual domain and focus area was assigned a numerical value. While there were 78 apps in the review, accounting for the multiple categorizations, this multi-select characterization yielded a total of 83 (55%) counts for one or more of the focus areas.

There were only six (8%) apps that utilized a theoretical or therapeutic framework underpinning their approach, including Cognitive Behavioral Therapy (CBT)^43^, Dialectic Behavioral Therapy (DBT)^44^, and Stages of Change/Transtheoretical Model^45^. Five of these six apps were focused on mental health.

Personalization was defined based on whether the healthbot app as a whole has tailored its content, interface, and functionality to users, including individual user-based or user category-based accommodations. Furthermore, methods of data collection for content personalization were evaluated^41^. Personalization features were only identified in 47 apps (60%), of which all required information drawn from users’ active participation. All of the 47 personalized apps employed individuated personalization. Forty-three of these (90%) apps personalized the content, and five (10%) personalized the user interface of the app. Examples of individuated content include the healthbot asking for the user’s name and addressing them by their name; or the healthbot asking for the user’s health condition and providing information pertinent to their health status. In addition to the content, some apps allowed for customization of the user interface by allowing the user to pick their preferred background color and image.

We also documented any additional engagement features the app contained. The most frequently included additional feature was the use of push notifications (20%) to remind the user to utilize the app. Table 1 lists the other additional features along with their frequencies. All of the apps were available as installable software via mobile devices and tablets and eleven of them (11%) were also accessible via a web browser on laptops, desktop computers, phones, and tablets. Very few apps provided any security-type features: nine (12%) included e-mail verification, three (4%) required text verification, one (1%) provided social media verification, and one (1%) required a password to access the app. Sixty-two apps (79%) did not contain any security elements including no requirements for a login or a password.

Information about data privacy was also limited and variable. Thirteen apps (16%) provided a medical disclaimer for use of their apps. Only ten apps (12%) stated that they were HIPAA compliant, and three (4%) were Child Online Privacy and Protection Act (COPPA)-compliant. Fifty-one apps (63%) did not have or mention any privacy elements.

### Geographic distribution

**Fig. 2:**
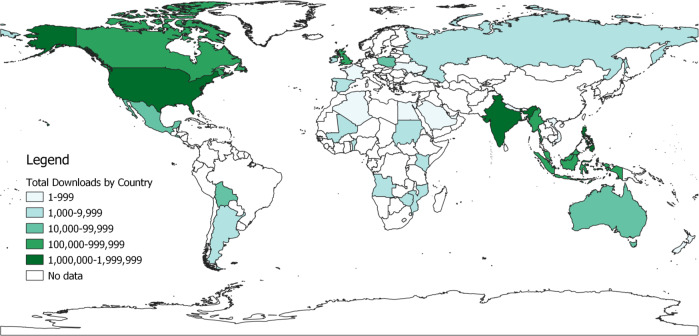
Geographic Distribution of Total Google Play Store Chatbot App Downloads, by Country. Source: UIA World Country Boundaries [2021]. Belgiu M., UNIGIS International Association, ArcGIS Hub.

For each app, data on the number of downloads were abstracted for five countries with the highest numbers of downloads over the previous 30 days. This feature was only available on the Google Play store for 22 apps. A total of 33 countries are represented in the map in Fig. 2. Chatbot apps were downloaded globally, including in several African and Asian countries with more limited smartphone penetration. The United States had the highest number of total downloads (~1.9 million downloads, 12 apps), followed by India (~1.4 million downloads, 13 apps) and the Philippines (~1.25 million downloads, 4 apps). Details on the number of downloads and app across the 33 countries are available in Appendix 2.

### NLP characteristics

Table 2 presents an overview of the characterizations of the apps’ NLP systems. Identifying and characterizing elements of NLP is challenging, as apps do not explicitly state their machine learning approach. We were able to determine the dialogue management system and the dialogue interaction method of the healthbot for 92% of apps. Dialogue management is the high-level design of how the healthbot will maintain the entire conversation while the dialogue interaction method is the way in which the user interacts with the system. While these choices are often tied together, e.g., finite-state and fixed input, we do see examples of finite-state dialogue management with the semantic parser interaction method. Ninety-six percent of apps employed a finite-state conversational design, indicating that users are taken through a flow of predetermined steps then provided with a response. One app was frame-based, which is better able to process user input even if it does not occur in a linear sequence (e.g., reinitiating a topic from further back in the conversation), and two were agent-based, which allows for more free-form and complex conversations. The majority (83%) had a fixed-input dialogue interaction method, indicating that the healthbot led the conversation flow. This was typically done by providing “button-push” options for user-indicated responses. Four apps utilized AI generation, indicating that the user could write two to three sentences to the healthbot and receive a potentially relevant response. Two apps (3%) utilized a basic parser, and one used a semantics parser (1%).

**Table 2:** Characterization of NLP system design.

Evaluation criteria	*n* (%)
*Dialogue interaction method* *(n* *=* *78)*	
AI generation	4 (5.1)
Fixed input	65 (83.3)
Basic parser	2 (2.6)
Semantic parser	1 (1.3)
Undetermined	6 (7.7)
*Dialogue management (n* = *78)*	
Finite state	69 (88.5)
Agent-based	2 (2.6)
Frame-based	1 (1.3)
Undetermined	6 (7.7)
*Dialogue initiative (n* = *78)*	
User	7 (9.0)
System	65 (83.3)
Mixed	1 (1.3)
Undetermined	5 (6.6)
*Input modality (n* = *87*^a^*)*	
Spoken	7 (8.0)
Written	75 (86.2)
Visual	3 (3.4)
Undetermined	2 (2.3)
*Output modality (n* = *88*^a^*)*	
Spoken	5 (5.7)
Written	76 (86.4)
Visual	5 (5.7)
Undetermined	2 (2.3)
*Task-oriented (n* = *78)*	
Yes	64 (82.1)
No	12 (15.4)
Undetermined	2 (2.6)

^a^Total sample size exceeds 78 because the chatbot can fulfill multiple categories.

We were able to identify the input and output modalities for 98% of apps. Input modality, or how the user interacts with the chatbot, was primarily text-based (96%), with seven apps (9%) allowing for spoken/verbal input, and three (4%) allowing for visual input. Visual input consisted of mood and food trackers that utilized emojis or GIFs. For the output modality, or how the chatbot interacts with the user, all accessible apps had a text-based interface (98%), with five apps (6%) also allowing spoken/verbal output, and six apps (8%) supporting visual output. Visual output, in this case, included the use of an embodied avatar with modified expressions in response to user input. Eighty-two percent of apps had a specific task for the user to focus on (i.e., entering symptoms).

## Discussion

We identified 78 healthbot apps commercially available on the Google Play and Apple iOS stores. Healthbot apps are being used across 33 countries, including some locations with more limited penetration of smartphones and 3G connectivity. The healthbots serve a range of functions including the provision of health education, assessment of symptoms, and assistance with tasks such as scheduling. Currently, most bots available on app stores are patient-facing and focus on the areas of primary care and mental health. Only six (8%) of apps included in the review had a theoretical/therapeutic underpinning for their approach. Two-thirds of the apps contained features to personalize the app content to each user based on data collected from them. Seventy-nine percent apps did not have any of the security features assessed and only 10 apps reported HIPAA compliance.

The proposed framework that facilitates an assessment of the elements of the NLP system design of the healthbots is a novel contribution of this work. Most healthbots rely on rule-based approaches and finite-state dialogue management. They mainly rely on directing the user through a predetermined path to provide a response. Most included apps were fixed-input, i.e., the healthbot primarily led the conversation through written input and output modalities. Another scoping review of mental healthbots also yielded similar findings, noting that most included healthbots were rule-based with a system-based dialogue initiative, and primarily written input and output modalities^33^. Assessing these aspects of the NLP system sheds light on the level of automation of the bots (i.e., the amount of communication that is driven by the bot based on learning from the user-specific data, versus the conversation that has been hard-coded as an algorithm). This has direct implications on the value of, and the risks associated with the use of, the healthbot apps. Healthbots that use NLP for automation can be user-led, respond to user input, build a better rapport with the user, and facilitate more engaged and effective person-centered care^9,41^. Conversely, when healthbots are driven by the NLP engine, they might also pose unique risks to the user^46,47^, especially in cases where they are expected to serve a function based on knowledge about the user, where an empathetic response might be needed. A 2019 study found that a decision-making algorithm used in healthcare was found to be racially biased, affecting millions of Black people in the United States^48^. An understanding of the NLP system design can help advance the knowledge on the safety measures needed for the clinical/public health use and recommendation of such apps.

Despite limitations in access to smartphones and 3G connectivity, our review highlights the growing use of chatbot apps in low- and middle-income countries. In such contexts, chatbots may fill a critical gap in access to health services. Whereas in high-income countries, healthbots may largely be a supplement to face-to-face clinical care, in contexts where there is a shortage of healthcare providers, they have a more critical function in triaging individuals presenting with symptoms and referring them to care, if necessary, thereby, reducing the burden on traditional healthcare services. Additionally, such bots also play an important role in providing counselling and social support to individuals who might suffer from conditions that may be stigmatized or have a shortage of skilled healthcare providers. Many of the apps reviewed were focused on mental health, as was seen in other reviews of health chatbots^9,27,30,33^.

Several areas for further development of such chatbot apps have been identified through this review. First, data privacy and security continue to be a significant and prevalent concern—especially when sharing potentially sensitive health-related data. A small percentage of apps included in our review noted any form of data privacy or security, namely via identity verification and use of HIPAA or COPAA. With the sensitive nature of the data, it is important for these health-related chatbot apps to be transparent about how they are ensuring the confidentiality and safety of the data that is being shared with them^49^. A review of mHealth apps recommended nine items to support data privacy and security, which included ensuring the patient has control over the data provided, password authentication to access the app, and privacy policy disclosures^50^. Second, most healthbots have written input. Development and testing of such chatbot apps with larger input options such as spoken, visual, will facilitate improvements in access and utility. Third, several chatbot apps claim the use of artificial intelligence and machine learning but provide no further details. Our assessment of the NLP system design was limited given the scarce reporting on these aspects. As apps increasingly use ML, for utility in the healthcare context, they will need to be systematically assessed to ensure the safety of target users. This raises a need for clearer reporting on aspects of the ML techniques incorporated. As healthbots evolve to use newer methods to improve usability, satisfaction, and engagement, so do new risks associated with the automation of the chat interface. Healthbot app creators should report on what type of safety and bias protection mechanisms are employed to mitigate potential harm to their users, explain potential harms and risks of using the healthbot app to the users, and regularly monitor and track these mechanisms. These risks should be included in an industry-standard such as the ISO/TS 25238^51^. It is also important for the system to be transparent regarding the recommendations and informational responses, and how they are generated^52^. The databases and algorithms that are used to program healthbots are not removed from bias, which can cause further harm to users if not accounted for. The framework presented in this paper can guide systematic assessments and documents of features of healthbots.

To our knowledge, our study is the first comprehensive review of healthbots that are commercially available on the Apple iOS store and Google Play stores. Laranjo et al. conducted a systematic review of 17 peer-reviewed articles^9^. This review highlighted promising results regarding the acceptability of healthbots for health, as well as the preference for finite-state (wherein users are guided through a series of predetermined steps in the chatbot interaction) and frame-based (wherein the chatbot asks user questions to determine the direction of the interaction) dialogue management systems^9^. Another review conducted by Montenegro et al. developed a taxonomy of healthbots related to health^32^. Both of these reviews focused on healthbots that were available in scientific literature only and did not include commercially available apps. Our study leverages and further develops the evaluative criteria developed by Laranjo et al. and Montenegro et al. to assess commercially available health apps^9,32^. Similar to our findings, existing reviews of healthbots reported the paucity of standardization metrics to evaluate such chatbots, which limits the ability to rigorously understand the effectiveness, user satisfaction and engagement, risks and harm caused by the chatbot, and potential for use^30^.

The findings of this review should be seen in the light of some limitations. First, we used IAB categories, classification parameters utilized by 42Matters; this relied on the correct classification of apps by 42Matters and might have resulted in the potential exclusion of relevant apps. Additionally, the use of healthbots in healthcare is a nascent field, and there is a limited amount of literature to compare our results. Furthermore, we were unable to extract data regarding the number of app downloads for the Apple iOS store, only the number of ratings. This resulted in the drawback of not being able to fully understand the geographic distribution of healthbots across both stores. These data are not intended to quantify the penetration of healthbots globally, but are presented to highlight the broad global reach of such interventions. Only 10% of the apps were screened by two reviewers. Another limitation stems from the fact that in-app purchases were not assessed; therefore, this review highlights features and functionality only of apps that are free to use. Lastly, our review is limited by the limitations in reporting on aspects of security, privacy and exact utilization of ML. While our research team assessed the NLP system design for each app by downloading and engaging with the bots, it is possible that certain aspects of the NLP system design were misclassified.

Our review suggests that healthbots, while potentially transformative in centering care around the user, are in a nascent state of development and require further research on development, automation, and adoption for a population-level health impact.

### Reporting summary

Further information on research design is available in the Nature Research Reporting Summary linked to this article.

## Supplementary information

Supplementary Information

Reporting summary

## Data Availability

Data can be made available upon request.
