# Supplementary material for: Health-focused conversational agents in person-centered care: a review of apps
Source: NPJ Digit Med. 2022 Feb 17;5:21. doi: 10.1038/s41746-022-00560-6 (PMC8854396; doi:10.1038/s41746-022-00560-6)
Supplement: Supplementary file 1 — Supplementary Information [file 41746_2022_560_MOESM1_ESM.pdf]

Supplementary Table 1: List of all apps analyzed

**Included Apps**			
**App**	**iOS**	**GP**	**Both**
Ada ,Äì your health companion	x		
Adent Health	x		
AfyaChats		x	
AI based Smart Doctor		x	
AI DOC		x	
AIDA - Ivy.ai Bot	x		
Analyze skin problems | AI Skin Symptom Checker		x	
Anesthesia Assistant	x		
Ask Mama		x	
Basic Emergency Care			x
BeCare Bot	x		
BeSerene | Mind Training and Stress Management App			x
Breathe for Asthma and COPD	x		
Brook Health Companion	x		
Careviz	x		
Coach Orange			x
Confidant Health	x		
COVID AI	x		
COVID-19	x		
Curable Pain Relief	x		

Diabcura: Personalised Diabetes Management		x	
DR Analyst		x	
Dr Lal PathLabs	x		
Dr.Galen - Health	x		
Driven Resilience App			x
Friendo ,Äì AI Virtual Friend	x		
Fudo: Picky Eating Help	x		
Gali Health	x		
GeneFAX	x		
Groove Health			x
GYANT - Pocket Doctor	x		
GYFT Baby			x
Haby App	x		
Healthily: The Self-Care App	x		
I need hugs		x	
InnerHour - Live Happier	x		
Joyup Genie		x	
Khoch		x	
Lafiya App - 24/7 Telehealth Anywhere Anytime		x	
LiveBetter			x
LiveMD - Talk to a Doctor		x	
Lotus - The Calming App	x		
MayaMD			x

Medic101		x	
Meela			x
Mindspa-4UR Mental Wellbeing	x		
Muse Health			x
myFetalLife	x		
MyHealthyGut: Guided Nutrition	x		
Nathealth VA			x
OneTapCare Travel Health	x		
OWL Cancer Survivor Platform			x
Ozee AI		x	
PAŞHAKEEM		x	
PocketFriend Digital Therapy	x		
QuitNow!	x		
Replika - My AI Friend	x		
Rosie	x		
RoundGlass Peace	x		
Sensely	x		
SimbiHealth		x	
SleepCoach	x		
Smoke Free +	x		
Sophie Bot		x	
Tara Smyles		x	
tellbeth			x
Thrive Nutrition Coach	x		
Tidda A.I		x	

TrackActive Me: Virtual Physio	x		
Velmio: Pregnancy Insights	x		
Venki - The Healthcare Chatbot		x	
Vik Breast			x
Woebot - Your Self-Care Expert	x		
Wysa: Mental Health Support			x
Youper - Feel your best			x
Zemedy - IBS, Gut Relief	x		
Zifcare			x
ZINI the Healthcare AI		x	

Supplementary Table 2: Total Number of Apps and Downloads by Country*

Continent	Country	Total Number of Apps, per Country	Name of Apps	Total Number of Downloads
Africa	Algeria	1	Pashakeem	32
Africa	Angola	1	Dr. Lal Pathlabs	5000
Africa	Benin	1	MayaMD	1280
Africa	Egypt	1	Pashakeem	924
Africa	Kenya	1	SophieBot	5000
Africa	Mali	1	MayaMD	1380
Africa	Mozambique	1	Dr. Lal Pathlabs	5000
Africa	Sudan	1	Dr. Lal Pathlabs	6000
Africa	Zimbabwe	2	Dr. Lal Pathlabs; MayaMD	6880
Asia	Indonesia	1	Wysa	105000
Asia	Nepal	1	MayaMD	680
Asia	Saudi Arabia	1	Pashakeem	19
Asia	United Arab Emirates	1	Pashakeem	19
Asia	Vietnam	1	ZINI the Healthcare AI	35
Asia	Malaysia	2	Replika; Wysa	255000
Asia	Myanmar	2	MayaMD; Youper	245740
Asia	Phillippines	4	Analyze skin problems | Skin Symptom Checker; Replika; Wysa; ZINI the Healthcare AI	1244110

Asia	India	13	Analyze skin problems | Skin Symptom Checker; BeSerene; Diabcura; Dr. Lal Pathlabs; LiveBetter; LiveMD: MindSpa-4UR Mental Wellbeing; Ozee AI; Replika; Wysa; Youper; Zifcare; ZINI the Healthcare AI	1395993
Australia	Australia	3	Curable Pain Relief; Driven Resilience App; LiveBetter	10552
Europe	France	1	VikBreast	100
Europe	Ireland	1	Curable Pain Relief	2300
Europe	Poland	1	Youper	48000
Europe	Spain	1	I need hugs	42500
Europe	Ukraine	1	MindSpa-4UR Mental Wellbeing	500
Europe	United Kingdom	8	Analyze skin problems | Skin Symptom Checker; Curable Pain Relief; Driven Resilience App; Healthily; I need hugs; LiveBetter; MindSpa-4UR Mental Wellbeing; Youper	658305
Europe/Asia	Russia	1	MindSpa-4UR Mental Wellbeing	8210
North America	El Salvador	1	Healthily	37000
North America	Mexico	2	Healthily; I need hugs	79750

North America	Canada	5	Analyze skin problems | Skin Symptom Checker; Curable Pain Relief; Driven Resilience App; Replika; ZINI the Healthcare AI	192639
North America	United States	12	Analyze skin problems | Skin Symptom Checker; Curable Pain Relief; Driven Resilience App; Groove Health; Healthily; I need hugs; LiveMD; MindSpa-4UR Mental Wellbeing; Replika; Wysa; Youper; ZINI the Healthcare AI	1875957
Oceania	New Zealand	1	Driven Resilience App	44
South America	Argentina	1	I need hugs	5450
South America	Bolivia	1	Healthily	39000

*Data was only able to be abstracted for apps available on the Google Play Store in July 2020. For each app, data on the number of downloads was abstracted for 5 countries with the highest number of downloads.
