# Supplementary material for: Health-focused conversational agents in person-centered care: a review of apps
Source: NPJ Digit Med. 2022 Feb 17;5:21. doi: 10.1038/s41746-022-00560-6 (PMC8854396; doi:10.1038/s41746-022-00560-6)
Supplement: Supplementary file 2 — Reporting summary [file 41746_2022_560_MOESM2_ESM.pdf]

nature portfolio

Corresponding author(s): Smisha Agarwal

Last updated by author(s): Dec 13, 2021

Reporting Summary

Nature Portfolio wishes to improve the reproducibility of the work that we publish. This form provides structure for consistency and transparency in reporting. For further information on Nature Portfolio policies, see our Editorial Policies and the Editorial Policy Checklist.

Statistics

For all statistical analyses, confirm that the following items are present in the figure legend, table legend, main text, or Methods section.

n/a	Confirmed	
☒	☐	The exact sample size (n) for each experimental group/condition, given as a discrete number and unit of measurement
☒	☐	A statement on whether measurements were taken from distinct samples or whether the same sample was measured repeatedly
☒	☐	The statistical test(s) used AND whether they are one- or two-sided<br>*Only common tests should be described solely by name; describe more complex techniques in the Methods section.*
☒	☐	A description of all covariates tested
☒	☐	A description of any assumptions or corrections, such as tests of normality and adjustment for multiple comparisons
☒	☐	A full description of the statistical parameters including central tendency (e.g. means) or other basic estimates (e.g. regression coefficient) AND variation (e.g. standard deviation) or associated estimates of uncertainty (e.g. confidence intervals)
☒	☐	For null hypothesis testing, the test statistic (e.g. F, t, r) with confidence intervals, effect sizes, degrees of freedom and P value noted<br>*Give P values as exact values whenever suitable.*
☒	☐	For Bayesian analysis, information on the choice of priors and Markov chain Monte Carlo settings
☒	☐	For hierarchical and complex designs, identification of the appropriate level for tests and full reporting of outcomes
☒	☐	Estimates of effect sizes (e.g. Cohen's d, Pearson's r), indicating how they were calculated

Our web collection on statistics for biologists contains articles on many of the points above.

Software and code

Policy information about availability of computer code

Data collection	Not applicable.
Data analysis	Not applicable.

For manuscripts utilizing custom algorithms or software that are central to the research but not yet described in published literature, software must be made available to editors and reviewers. We strongly encourage code deposition in a community repository (e.g. GitHub). See the Nature Portfolio guidelines for submitting code & software for further information.

Data

Policy information about availability of data

All manuscripts must include a data availability statement. This statement should provide the following information, where applicable:

- Accession codes, unique identifiers, or web links for publicly available datasets
- A description of any restrictions on data availability
- For clinical datasets or third party data, please ensure that the statement adheres to our policy

Data can be made available upon request.

Field-specific reporting

Please select the one below that is the best fit for your research. If you are not sure, read the appropriate sections before making your selection.

☐ Life sciences ☒ Behavioural & social sciences ☐ Ecological, evolutionary & environmental sciences

For a reference copy of the document with all sections, see nature.com/documents/nr-reporting-summary-flat.pdf

Behavioural & social sciences study design

All studies must disclose on these points even when the disclosure is negative.

Study description	A review of health apps that include a chatbot feature ("healthbots") that are available on the Google Play or Apple iOS store.
Research sample	Apps that were health-related, had an embedded text-based conversational agent, available for free public download through the Google Play or Apple iOS store, available in English, and facilitated a bidirectional (two-way) conversation between the app and the user were included in the research sample.
Sampling strategy	All healthbots that met the eligibility criteria were included.
Data collection	We ran the search on 42Matters, a proprietary software database that collects app intelligence and mobile tracking data, to identify potential healthbot apps. Research team screened the healthbot apps and determined if they were eligible for inclusion. Several elements of data were collected: app title, package name, language, number of downloads (Google Play only), average rating, number of ratings, availability of in-app purchases, date of last update, release date (Apple iOS only), use of IBM Watson software, Google sign-in (Apple iOS only), and Facebook sign-in (Apple iOS only). Utilization of Android permissions for Bluetooth, body sensors, phone calls, camera, accounts access, and internet, as well as country-level downloads were also extracted from Google Play store only.
Timing	Conducted search in June and July 2020.
Data exclusions	Healthbot apps were excluded only if they did not meet the eligibility criteria.
Non-participation	Not applicable.
Randomization	Not applicable.

Reporting for specific materials, systems and methods

We require information from authors about some types of materials, experimental systems and methods used in many studies. Here, indicate whether each material, system or method listed is relevant to your study. If you are not sure if a list item applies to your research, read the appropriate section before selecting a response.

Materials & experimental systems

n/a	Involved in the study	
☒	☐	Antibodies
☒	☐	Eukaryotic cell lines
☒	☐	Palaeontology and archaeology
☒	☐	Animals and other organisms
☒	☐	Human research participants
☒	☐	Clinical data
☒	☐	Dual use research of concern

Methods

n/a	Involved in the study	
☒	☐	ChIP-seq
☒	☐	Flow cytometry
☒	☐	MRI-based neuroimaging
